# Analysis of Factors Influencing Public Behavior Decision Making: Under Mass Incidents

**DOI:** 10.3389/fpsyg.2022.848075

**Published:** 2022-05-16

**Authors:** Rui Shi, Chang Liu, Nida Gull

**Affiliations:** School of Economics and Management, Yanshan University, Qinhuangdao, China

**Keywords:** mass incidents, behavior decision-making, influencing factors, emotional factors, structural equation (SEM)

## Abstract

Most mass incidents are created by economic or social concerns brought on by fast socioeconomic change and poor local government. The number of mass occurrences in China has significantly increased in recent years, putting the country’s steady growth and public behavior decision-making in harm. We examine the factors that influence public behavior decision-making in the following significant factors, contributing to the development of effective prevention and response strategies. The structural equation (SEM) approach is used to analyze the main determinants influencing public behavioral decisions in the aftermath of mass incidents using surveys of a large population. The finding shows that media plays a mediating role in the relationship between mass occurrences and influencing factors impacting public emotion. The direct and indirect effects of public behavior decision-making and its role increasingly social changes as things happen, government credibility, media plays mediating role in public emotional factors. All directly impact public behavior decision-making, while emotional factors have an indirect impact via media intermediaries. The escalation of public behavior decisions is seen as a result of structural transmission and the increase of dynamic as well as other factors.

## Introduction

Since the late 1970s, when China began its socioeconomic changes, social demonstrations have trailed the process like inseparable shadows every step of the way. There have always been positives and negatives when money and power are divided (Chi et al., 2006). The more profound the shifts get, the more social unrest emerges. Tax riots, land, and labor conflicts, environmental protests, and racial confrontations are all examples of mass social demonstrations (McGeehan et al., 2006). To be sure, these mass incidents highlight the hardships and problems that come with China’s development. They are, to some extent, typical symptoms of any culture undergoing significant social and economic change. However, persistent and widespread civil disturbance (Vermuyten et al., 2016) may be the catalyst for regime change. Mass incidents are thought to be expressions of deep societal discontent and barometers for regime stability, social behavior researchers have transformed their attention to studying social protest in contemporary China (Wang et al., 2019).

Mass public emergencies have been accompanied by a rapid increase in China’s social transition in recent years, causing chaos in the country’s social order and public safety (O’Neil et al., 2008); Because of the complexities of the effects and root causes of large-scale public emergencies, as well as the diversity of stakeholders (McGeehan et al., 2006). Due to the apparent large scale of incidents, their short duration, and the multi-channel dissemination of true and false information, the variety and ferocity of conflicts (Liu et al., 2019), public action decisions have been made (Xing et al., 2019). This has appropriately made preventing and responding to major public emergencies more difficult. Wang et al. (2019) have identified the critical factors that influence public behavior decision-making in times of public emergency. To better understand group emergency mechanisms and assist in the rational development of group disaster prevention and response methods.

Xing et al. (2019) have conducted to determine the components that influence public behavior decision-making in times of public emergency (Ragin, 2009), identifying several critical factors regarding group emergencies’ impact on public behavior. Purohit et al. (2016), group conflict behavior is caused by a conflict between societal resources and individual demands and beliefs. Participants in group behavior respond to certain triggering conditions, and social institutions adopt actions (Outten and Schmitt, 2015). According to Maitner et al. (2006), the degree of group identity affects the psychological changes of group members and hence group behavior intentions. The psychological factors affecting group members influence collective behavior decisions and public decision-making (Outten and Schmitt, 2015). The mechanism by which emotional factors determine collective behavior intention is the basic process of group emotion communication (Kauffmann and Kikuchi, 2013).

Watson (1978) discovered the active involvement of social media in relieving the mood and psychology of the public. Empirical study demonstrates that three things influence group behavior: their psychology, behavior, and the dominant social knowledge (Liu et al., 2019). Through grounded theory, Shen et al. (2018), have revealed that fairness is a critical component of the three variables of power, resistance, and drive. Ma and Wang, 2009 have shown that when unexpected large-scale public events occur, game theory and behavioral operation management theory may be used to explain the cooperative evolution process of cooperation and conflict between groups and organizations. Qin et al. (2021), have developed a “scenario-response” system for government crisis decision-making from the government’s perspective and offered a helpful contingency plan for policymakers. Wang et al. (2019) stated that creating an emergency response system has a significant impact on developing prevention and control events; failing to do so could result in severe consequences. Based on rooted theory, explained that the four variables of the specific situation, social structure, group action, and psychology, all affect group public emergencies (Cai, 2008).

The existing literature focuses mainly on the variables driving mass emergencies, with little research on the factors influencing public policy decisions in group public emergencies (Rudd et al., 2012). As a result, this research should help build public awareness in large-scale public situations. This study aims to investigate the factors that influence public behavior decision-making during mass emergencies. Based on (the S–O–R decision model), group emergencies-inducing variables are the primary driving force behind mass emergency participation. On the other hand, mass emergencies can cause the individual to produce emotions, which is another contributing factor, and various levels of emotion affect individual decision-making behavior. In the relationship between influencing factors and decision-making, government credibility is a mediator. Furthermore, media intermediaries play a mediating role in the relationship between influencing factors and decision-making (Wang et al., 2019). As a result, this study investigates the association between these variables in order to understand the elements that influence public behavior decision-making and, as a result, formulate appropriate management measures to prevent mass catastrophes. The main theoretical contribution of this research is the relationship between the hypothesis that affects behavioral decision-making, the conceptual model being built, prior research combined with representative shared community group emergencies, the measuring questionnaire design, and data collection. The multi-factor structure equation model of public conduct decision-making in group public emergencies is based on structural equation theory. The constant research of the mechanism of interaction between different elements and behavioral decision-making during public group emergencies once the hypothesis has been confirmed.

## Literature Review and Research Hypotheses

### Predisposing Factors

In terms of group structure and function, a social group can be defined as a group of two or more people who have common goals and expectations and can work together toward a common goal (Ma and Wang, 2009; Purohit et al., 2016). The parties or groups of third-party public emergencies come from different organizations or groups. Even if the participants or related employees have different backgrounds, educational experiences, social cognition, personality preferences, and ways of thinking, they have common appeal and collective identity (Falatoonitoosi et al., 2013; Shen et al., 2018). From the perspective of group size and group behavior, group members want more people to join once a group is formed. The more people participate, the more likely people share emotions (Årstad and Aven, 2017). The higher the group’s confidence in carrying out collective action, and the stronger the motivation, the “many strength of the people,” the larger the group size. When a large number of people participate in a demonstration, bystanders have a better chance of succeeding (Basir et al., 2018). Individuals who are less interested or less engaged than others are encouraged by this collective power, demonstrating that the size of the group can demonstrate collective strength. Conflict is an important variable in the public decision-making process of mass public emergencies from the standpoint of justice and interest expectation. China’s social economy has grown quickly since reform and opening ups. Income, education, medical care, and social security have all showed significant inequalities due to disparities in development between regions, urban and rural areas, and ethnic groupings. The difference between urban and rural communities is widening, and some local government officials are dishonest and inept, and power and money collide, causing considerable harm to people’s interests (Karakitapoğlu-Aygün et al., 2020). With the influx of Western thoughts, the people’s self-interest and material culture needs have become more intense, making the inner sense of injustice stronger and stronger (Zio, 2016; Gitelman et al., 2017). In recent years, people’s awareness of their rights, as well as their sense of equality, has increased. When the government fails to meet the people’s inner interests and expectations, they perceive that the distribution of interests is unbalanced, and conflicts of interest between the government and the people emerge quickly, moving from passive acceptance to initiative, resulting in mass public emergencies.

### Government Credibility Factors

Social conflict, according to the notion of social conflict, is a manifestation of group behavior. Conflict arises from the fight for finite resources like as rights and authority, and it is managed by diverse organizations and groups to maintain social order (Li, 2021). The Chinese government is the most powerful force in the country when it comes to maintaining social order. The government is the most authoritative institutional body, regardless of popular social rights (Launder and Perry, 2014). Several conflicts of interest distribution are strong in the face of substantial public emergencies. The government fails to promptly provide the public with a reasonable and satisfying solution (Xie and Liu, 2019). People have lost faith in the government in that event, and the government’s credibility and authority suffered as a result. When normal and legitimate avenues fail to solve people’s problems, they turn to illegal channels like visits, demonstrations, rallies, sit-ins, and to satisfy or sustain critical interests. Such large public emergencies will unavoidably reach an unforeseen stage, with direct face-to-face disputes between the government and the public endangering social stability (Alsharif et al., 2021).

### Public Emotional Factors

Emotion is a well-organized, profound, and ever-changing state of consciousness (Xing et al., 2019). By recording the emotions of others, people can perceive changes in the state of their neighbors. Falatoonitoosi et al. (2013) founders of group psychology and social psychology, observed that group emotions’ mutual infection influences group behavior choice. Individual emotions will quickly infect (Chi et al., 2006), disseminate and spread in the group, generate emotional outbursts, sparking a chain reaction, and provoke more significant mass events. Furthermore, events involving prominent personalities or authoritative figures may be involved (Outten and Schmitt, 2015). They speak with a stronger voice and have more credibility than the average people (Vermuyten et al., 2016). For the people, their emotions and behaviors have symbolic and leadership value. These significant figures or authoritative figures have acted as “leaders” in the event, particularly in large-scale public emergencies where the public can easily become engulfed in a herd mentality. Individual, collective, and key individual emotions impact group public emergency decision-making (Wang et al., 2019).

### Media Intermediary Factors

When public catastrophes occur, news media, internet media, and other media are all involved due to modern networks and information technology (Falatoonitoosi et al., 2013). They play a role in the real-time dissemination of news, promoting event development and social progress, and maintaining social order. Traditional media (television, news, radio, newspapers, etc.) and online new media (WeChat, Weibo, blog, live APP, etc.) are the two types of media (Falatoonitoosi et al., 2013). After a public emergency, the media will report on the scene as soon as possible and disseminate vital information to the public in the shortest period possible (Song and Xin, 2016). The advantage of the new network media is that people are no longer passive recipients of information; instead, everyone can become a publisher and disseminator of information (Toyama and Hayashi, 2021). Moreover, during large-scale public emergencies, the public is prone to negative feelings such as terror and rage, and they are eager to learn the truth about the situation. As a result, they have actively seek information on the occurrence via various media sources. On the other hand, as event participants, they may not be in a good position to speak directly with government officials (Falatoonitoosi et al., 2013). Media intermediaries must serve as a link between the government and the general public in order to discuss and resolve crucial issues (Toyama and Hayashi, 2021). The public has choose more media help when it encounters group public emergencies, or publish and disseminate based on the online media platform, because the media’s positioning not only guides public opinion, but also because the public will choose more media help when it encounters group public emergencies, the media and public opinion have a two-way influence (Hou and Xu, 2021).

### Public Behavioral Decision Factors

Due to disparities in their experiences, education, and social cognition, individuals’ knowledge and comprehension of the same episode varied (LeBlanc et al., 2015). Public behavior decision-making can be loosely split into three sorts following huge public emergencies (Xie and Liu, 2019). The first is to confront the powerful groups directly and choose to fight or resist; the second is to choose silence, not to participate, or to compromise or cooperate; and the third is to choose to assist and coordinate one of the parties’ altruistic action (Wei et al., 2014).

**FIGURE 1:**
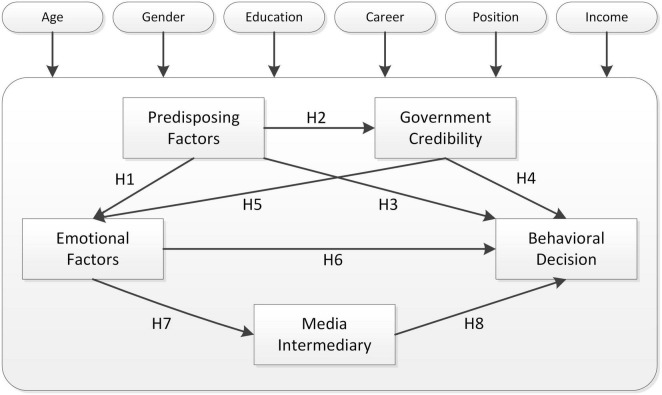
Multi-factor theoretical model of sentiment and behavior in public emergencies.

In summary, group structure, group size, group behavior, interest expectation, and fairness are the main predisposing factors for group public behavior; emotional factors include individual emotions, group emotions. The person’s emotions; government credibility factors include government credibility and legal binding; and media mediation factors include traditional media and online new media. As indicated in Figure 1, the core conceptual model is built around the relationship between the multi-factors of mass public sentiment and behavioral impacts. The following research hypotheses are offered in association with the conceptual model:

H1: Predisposing factors have a positive impact on public emotional factors.

H2: Predisposing factors have a positive impact on government credibility factors.

H3: Predisposing factors have a positive impact on public behavioral decisions.

H4: Government credibility factors have a positive impact on public behavioral decisions.

H5: Government credibility factors have a positive impact on public emotional factors.

H6: Public emotional factors have a positive impact on public behavioral decisions.

H7: Public emotional factors have a positive impact on media intermediary factors.

H8: Media intermediary factors have a positive impact on public behavioral decisions.

## Methodology

### Sample and Questionnaire Development

Previous research on questionnaire design can be used with existing forming scales. In view of the fact that there are few empirical researches on mass public emergencies in the current theory, this chapter is based on domestic and foreign related achievements and theories, and the “employee collective strikes due to the company’s arrears of wages” as the empirical case background of public emergencies. Conduct a questionnaire design. Since the variables identified in the conceptual model are abstract and not directly observable latent variables, it is necessary to design corresponding indicators for latent variables to form measurable observed variables. Determining the variables related to the multi-factor impact of public sentiment and public behavior in public emergencies: the dependent variable is the behavioral decision of the public in the public emergency of the group, mainly refers to the struggle and resistance, the compromise and cooperation behavior, the altruistic behavior; Variables are predisposing factors, public factors, government credibility and media intermediaries of public behaviors in public emergencies. Using the Likert five-level scoring method to score the questionnaire, a proposition is given, asking the respondent to indicate the attitude, 1 point is expressed as “completely non-conformity,” 2 points means “comparatively inconsistent,” and 3 points means “not met.” Obviously, 4 points means “comparable,” and 5 points means “very consistent.” The preliminary design questionnaire consists of 22 survey items, which are composed of the following parts: (1) Questionnaire guidance: Explain the purpose and requirements of the investigation to the respondent in order to understand the precautions of the investigation. (2) For the questionnaire survey on the factors influencing the public behavior decision-making of public emergencies, the questionnaire designed a total of 15 questions, corresponding to the Q1–Q15 questions. (3) The basic information of the respondents: Q16 age, Q17 gender, Q18 education level, Q19 employment status, Q20 occupation, Q21 position, and Q22 income.

The subjects were open to various levels of population, and the seven variables related to the basic information of the survey were analyzed separately. Fifty pre-experiment data were distributed in the early stage, and the pre-experiment data collected was analyzed by SPSS 25V. The results showed that the consistency coefficient of the questionnaire Cronbach’s was 0.912 > 0.7, indicating that the questionnaire has good internal consistency. The KMO coefficient is 0.851 greater than 0.5, the Bartlett spherical test *p*-value is less than 0.05, and the total variance explained is 79.96%. Then, based on the results of factor analysis, feedback from the survey respondents and interviews and guidance from experts in relevant fields, the variables and observation variables of the questionnaires were appropriately adjusted and modified to form the final formal questionnaire which indicated in Appendix.

## Data Analysis and Hypothesis Testing

### Sample and Data Collection

The questionnaire was distributed through the Internet Professional Questionnaire Website Questionnaire^1^. The principle of selecting respondents is the hierarchy and diversification of occupations, age groups, and educational backgrounds to ensure the consistency and effectiveness of public assessment of public emergencies. The questionnaire survey involved nearly 20 provinces across the country, 294 questionnaires were collected, 15 invalid questionnaires were removed, and 279 valid questionnaires were finally obtained. The recovery rate of the questionnaire was 97.38%. Table 1 shows the age, sex, education level, employment status, occupation, position, and monthly income of 279 samples. It can be seen that in terms of age, the youth (under 30 years old) and the strong (31–40 years old) account for a large proportion of the total sample size, which is in line with the general characteristics of the age group of the group public emergency participation; in terms of gender, the basic ratio of male to female In terms of education level, university education has the highest proportion; in terms of employment status, the number of employed people is the highest; occupation is mostly workers and employees; income is below 5,000 RMB.

**TABLE 1:** Basic characteristics of the sample.

Characteristics	Classification	Number of people	Proportion (%)
Age	20–	3	1.1
	21–30	94	33.7
	31–40	135	48.4
	41–50	35	12.5
	51–60	11	3.9
	60+	1	0.4
Gender	Male	124	44.4
	Female	155	55.6
Education	High school education below	78	28.0
	University degree or above	201	72.0
Employment status	Employment	253	90.7
	Unemployed or retired	26	9.3
Career	Workers or employees	137	49.1
	Farmer	10	3.6
	Small private business	31	11.1
	Student	36	12.9
	Civil servant	11	3.9
	Teacher	29	10.4
	Soldier	7	2.5
	Medical staff	4	1.4
	Others	14	5
Monthly income	3000–	109	39.1
	3001–5000	98	35.1
	5000+	72	25.8
			

### Descriptive Statistical Analysis

The maximum, minimum, mean, variance, standard deviation, skewness, and kurtosis of the observed values of each observed variable were analyzed. According to the absolute value of the kurtosis is not more than 8, the absolute value of the skewness is not more than 3, the sample basically obeys the normal distribution. The mean value of the sample is between (3.03 and 4.11), indicating that the respondents’ acceptance of the observed variable indicators is at the upper-middle level.

### Reliability Test

Reliability of data and is a measure of the consistency and stability of data. Reliability generally reflects the relationship between internal topics, and the higher the reliability, the better the stability of the observations (Arbuckle, 2010). This study used the internal consistency coefficient Cronbach’s coefficient to test the reliability of the questionnaire. The coefficient has a value between (0 and 1). The larger the value, the higher the internal consistency of the observed variable. Hair et al. (2014) believe that the coefficient is preferably higher than 0.70. The internal consistency test of the recovered sample data was carried out by SPSS.25V (Preacher and Hayes, 2004), and the total coefficient of the questionnaire was 0.852, and the coefficients of each latent variable were all above 0.7, indicating that the internal consistency of the index is high, which is suitable for the next analysis. See Table 2 for details.

**TABLE 2:** Cronbach’s reliability coefficient table for each variable.

Variable	C-α	Items	*CR*
Predisposing factors	0.821	5	0.852
Emotional factor	0.852	3	
Government credibility	0.713	2	
Behavior decision	0.734	3	
Media intermediary	0.853	2	

#### Validity Test

Validity is the degree of closeness between the observed result and the target. The validity analysis of this study mainly includes content validity and structural validity. The content validity is used to measure the adaptability between the measurement target and the measurement content. After the questionnaire design, the relevant items are judged, screened by relevant experts, and then corrected to meet the measurement objectives and requirements. Then pre-experiment is carried out to verify that the experimental results have met the relevant standards, and finally form a formal questionnaire, which can be considered to meet the requirements of the content in terms of content validity.

The rotated component matrix is shown in Table 3. The scores of each item are greater than 0.4, indicating that the observed variables can well explain the commonality of the latent variables. From the results, the items included in component 1 are Q5, Q6, Q4, Q7, and Q8, the items included in component 2 are Q2, Q1, and Q3, and the items included in component 3 (behavior decision) are Q11, Q12, and Q13. The items included in component 4 are Q14 and Q15, and the items included in component 5 are Q9 and Q10. Subsequently, the components obtained by factor analysis were named. Component 1 is the predisposing factor, component 2 is the emotional factor, component 3 is the behavioral decision, component 4 is the media intermediary factor, and component 5 is the government credibility factor.

**TABLE 3:** Cross loading component matrix.

	Composition				
Questions	Composition1	Composition2	Composition3	Composition4	Composition5
Q5	0.797	–	–	–	–
Q6	0.771	–	–	–	–
Q4	0.730	–	–	–	–
Q7	0.679	–	–	–	–
Q8	0.661	–	–	–	–
Q2	–	0.862	–	–	–
Q1	–	0.840	–	–	–
Q3	–	0.783	–	–	–
Q13	–	–	0.805	–	–
Q12	–	–	0.767	–	–
Q11	–	–	0.754	–	–
Q15	–	–	–	0.911	–
Q14	–	–	–	0.897	–
Q10	–	–	–	–	0.868
Q9	–	–	–	–	0.820

## Model Test and Result Analysis

**FIGURE 2:**
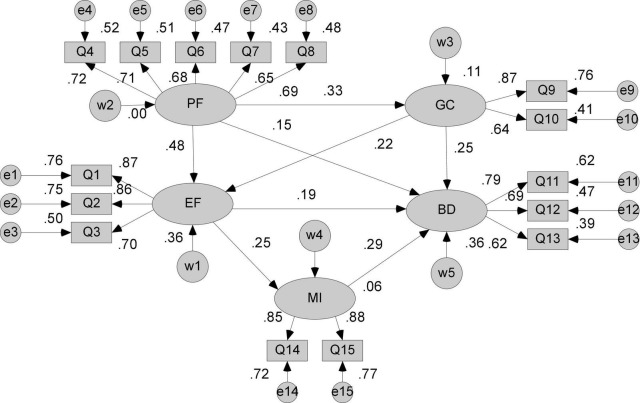
Initial model.

According to the basic assumptions and concept diagrams above, the structural equation model is drawn in AMOS 24.0 software, and the sample data Q1–Q15 is correlated, and the operation is performed by the maximum likelihood method. The result is shown in Figure 2. The path coefficient significance test is then judged based on the value of the critical ratio (CR; Asghar et al., 2020, 2021). If the absolute value of CR is greater than 1.96, it means that the estimated value *P* is significant at the level of 0.05. If the absolute value of CR is greater than 2.58, it indicates that the estimated value *P* is significant at the level of 0.01, and the operation results are shown in Table 4. It can be seen from the results that there is a path that does not reach a significant level, that is, the direct influence of the public emergency triggering factors on behavioral decision-making is not obvious, and the path needs to be deleted.

**TABLE 4:** Path coefficient estimation and parameter significance of the initial model.

			Estimate	SE	CR	*P*
Government credibility	←	Predisposing factors	0.429	0.098	4.380	***
Emotional factors	←	Predisposing factors	0.497	0.081	6.154	***
Emotional factors	←	Government credibility	0.177	0.060	2.941	0.003
Media intermediary	←	Emotional factors	0.305	0.086	3.540	***
Behavioral decision	←	Media intermediary	0.232	0.058	3.996	***
Behavioral decision	←	Emotional factors	0.187	0.087	2.144	0.032
Behavioral decision	←	Government credibility	0.195	0.066	2.933	0.003
Behavioral decision	←	Predisposing factors	0.152	0.087	1.751	0.080
Q4	←	Predisposing factors	1.000			
Q5	←	Predisposing factors	1.104	0.106	10.460	***
Q6	←	Predisposing factors	0.956	0.095	10.076	***
Q7	←	Predisposing factors	0.965	0.100	9.696	***
Q8	←	Predisposing factors	1.010	0.099	10.230	***
Q9	←	Government credibility	1.000			
Q10	←	Government credibility	0.795	0.142	5.615	***
Q15	←	Media intermediary	1.000			
Q14	←	Media intermediary	0.953	0.132	7.211	***
Q3	←	Emotional factors	1.000			
Q2	←	Emotional factors	1.173	0.092	12.792	***
Q1	←	Emotional factors	1.215	0.095	12.828	***
Q11	←	Behavioral decision	1.000			
Q12	←	Behavioral decision	1.092	0.118	9.263	***
Q13	←	Behavioral decision	0.872	0.101	8.680	***

***P < 0.001 (two tailed).

In order to make the model good fit, the initial model should be modified appropriately and adjusted, and the revised model should be more reasonable, complete, clear and fully explained. In this study, the correction index is mainly referred to the modification indices provided by AMOS software. When selecting the correction index, the larger of the modified index values is selected first because the modified index value indicates that when the fixed parameter is changed to the free parameter the entire model is squared reduced value (Xiaolong et al., 2021). That is to say, the larger the correction value, the larger the chi-square that can be reduced, but there is no fixed standard. Some studies believe that the correction index is higher than 3.84 and needs to be corrected, and other studies suggest that the correction is higher than 5. If the correction index of the model is less than 3.84, it indicates that the intrinsic quality of the model has sequence error. If the correction index is too large, it suggests the parameter. The adaptation situation is not good.

From the results of the revised index, the MI value of e6 and e13 is 10.899, which indicates that increasing the negative covariation relationship between these two items (group behavior and compromise or cooperative behavior) can make the chi-square of the structural equation model Will decrease. Studies have shown that in the case of mass public emergencies, the herd phenomenon is highly prone to occur, and the herd phenomenon can be explained by cognitive dissonance theory. Cognitive dissonance theory refers to individuals who recognize conflicts or conflicts between their attitudes or between attitudes and behaviors. That is to say, in the group behavior decision-making situation, the individual realizes that there is a contradiction or conflict between his behavior or attitude and the group behavior or attitude. This state is very likely to cause individual psychological tension and anxiety, and generate corresponding emotions. Emotions and herd behavior are inseparable. Therefore, increasing the e6 and e13 paths can be reasonably explained and theoretically supported.

The coefficient estimation and parameter saliency of the structural equation model obtained after re-estimation are shown in Table 5. The path coefficient CR and the significant *P* value satisfy the significance test requirements.

**TABLE 5:** Corrected coefficient estimates and parameter significance.

			Estimate	SE	CR	*P*
Government credibility	←	Predisposing factors	0.430	0.098	4.406	***
Emotional factors	←	Predisposing factors	0.506	0.081	6.232	***
Emotional factors	←	Government credibility	0.177	0.061	2.922	0.003
Behavioral decision	←	Media intermediary	0.307	0.086	3.563	***
Behavioral decision	←	Emotional factors	0.259	0.075	3.437	***
Behavioral decision	←	Government credibility	0.215	0.067	3.195	0.001
Q4	←	Predisposing factors	1.000			
Q5	←	Predisposing factors	1.103	0.106	10.452	***
Q6	←	Predisposing factors	0.953	0.094	10.155	***
Q7	←	Predisposing factors	0.962	0.990	9.669	***
Q8	←	Predisposing factors	1.010	0.900	10.234	***
Q9	←	Government credibility	1.000			
Q10	←	Government credibility	0.813	0.140	5.792	***
Q15	←	Media intermediary	1.000			
Q14	←	Media intermediary	0.938	0.125	7.485	***
Q3	←	Emotional factors	1.000			
Q2	←	Emotional factors	1.172	0.091	12.823	***
Q1	←	Emotional factors	1.211	0.094	12.846	***
Q11	←	Behavioral decision	1.000	0		
Q12	←	Behavioral decision	1.111	0.118	9.417	***
Q13	←	Behavioral decision	0.892	0.100	8.920	***

***P < 0.001 (two tailed).

The fitting results of the modified structural equation model are shown in Table 6, and all the fitting indices satisfy the standard. Therefore, the revision process of the model is completed, and the revised model is ideal.

**TABLE 6:** The results of the model fit.

	Model fit index	Standard	Model results	Status
Good fit index	CMIN/DF	<5	1.752	Accepted
	RMSEA	<0.08	0.052	Accepted
	GFI	>0.9	0.936	Accepted
Parsimonious fit index	PNFI	>0.5	0.715	Accepted
	PGFI	>0.5	0.640	Accepted
	AGFI	>0.9	0.907	Accepted
Value-added fitting index	CFI	>0.9	0.961	Accepted
	NFI	>0.9	0.915	Accepted
	IFI	>0.9	0.962	Accepted
	TLI	>0.9	0.950	Accepted

**FIGURE 3:**
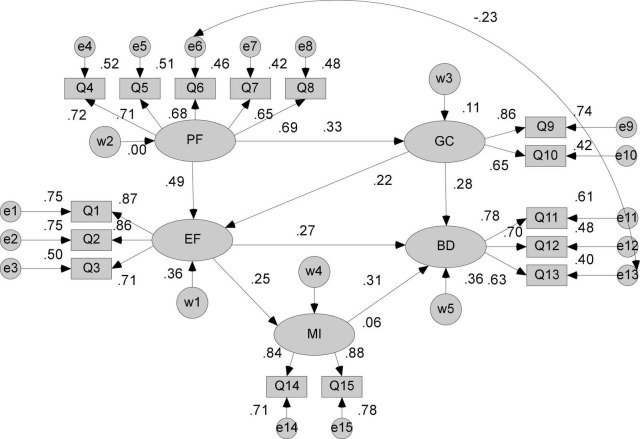
The revised structural equation model.

Figure 3 is the model of the optimal structural equation model finally determined for this study.

## Discussion and Conclusion

This research is based on observed variables (inducing elements, government credibility factors, media intermediaries) as well as emotional components obtained from public behavior judgments affecting group public emergencies. We will gather real data, investigate the relationship between independent and dependent variable factors, develop a structural equation model of the factors influencing public behavior decision-making during public emergencies, and ensure that the model fits well after reasonable model correction through research. The findings indicate that group public emergency triggering factors have a positive effect on emotional factors, with a path coefficient of (*b* = 0.49, *p* = 0.001); group public emergency triggering factors have a positive effect on government credibility, with a path coefficient of (*b* = 0.33, *p* = 0.001); and government public trust in group public emergencies has a positive effect on behavior. Influence, and the path coefficient is (*b* = 0.22, *p* = 0.001); the mass public sentiment factor has a positive effect on behavioral decision-making, and the path coefficient is (*b* = 0.27, *p* = 0.001); and the collective public event emotional factor has a positive effect on behavioral decision-making, and the path coefficient is (*b* = 0.27, *p* = 0.001). The media intermediate has a beneficial effect, as evidenced by the (*b* = 0.25, *p* = 0.001) path coefficient. The media intermediary had a positive effect on the group’s decision to engage in public emergency behavior, with a path coefficient of (*b* = 0.31, *p* = 0.001). The path diagram of the structural equation model can be used to determine the structural covariation relationship between the variables, as well as the direct and indirect effects and total effects between the latent variables.

The role of influencing factors in the complex link between public conduct and decision-making in times of public emergency. The model indicates that the predisposing elements are the cause variables of public emergencies in groups, while also acting on emotional components. Direct effects have a value of *b* = 0.49, which is positively significant (*p* = 0.001). Following that, emotional components had a direct effect on behavioral decision-making, with a (*b* = 0.27) effect coefficient and a positive effect (*p* = 0.001). Additionally, the media intermediary can be employed as a mediator variable to influence the behavioral decision (*b* = 0.142) in an indirect manner, and it has a substantial positive effect (*p* = 0.001). Following a sequence of mediator variables, the overall effect coefficient of the influencing factors on the link between public emergency mood and conduct was (*b* = 0.289). The major source of mass public emergencies is influencing situations, which expose individuals to severe internal and external stimuli. Internally, their desire for justice and expectations of interests serve as the “internal driver” of their activities, with external cues such as groups of a certain size or “similar type with shared viewpoint or background” supplementing them. Individuals, and thus collective conduct, which provides participants with a sense of collective identity and belonging, should act in a similar manner to others, and even create herd behavior, in order to aid the event’s evolution. Moreover, influencing factors do not have a direct effect on behavioral decision-making, but rather through a series of mediating variables, most notably emotions, according to the study. The role of the influencing factors steadily reduces as the event occurs. People gradually transition from a sense of inner injustice or dissatisfaction to a range of emotional outbursts throughout the event and thus become exposed to the effect of emotions decisions are frequently irrational.

The role of government credibility in the multi-factor relationship of public behavior decision-making under mass public events. The government’s credibility has a direct effect coefficient of (*b* = 0.28) on behavioral decision-making and is positively significant (*P* < 0.001), that is, when the government’s credibility is lower (the higher the score), the public participation group The higher the probability of a sexual emergency, the easier it is to happen. Since the government plays a major role in the prevention, control and governance of public emergencies, it raises the government’s credibility and improves the corresponding legal operation mechanism and legislative work, expands the popularization of law and publicity, increases the public’s dependence on the government, and deepens the political economy. The reform of the system allows the people to truly feel the benefits of economic construction and eliminate the unfairness of the people, which can greatly reduce the probability of public emergencies. In addition to the direct effect, the government’s credibility indirectly affects behavioral decision-making through emotional factors. The indirect effect coefficient is (*b* = 0.077), and it has a positive impact (*P* < 0.001). After a series of mediator variables, the government’s credibility is obtained. The overall effect coefficient in the relationship between sentiment and behavioral impact of mass public events is (*b* = 0.354). It can be seen that improving the government’s credibility, establishing and perfecting the people’s fairness appeal channels can effectively prevent group public emergencies.

The role of media intermediaries in the multi-factor relationship of public behavior decision-making under mass public events. From the model, media mediation has a direct effect on behavioral decision-making, with a path coefficient of (*b* = 0.31) and positive impact (*P* < 0.001). It can be seen that with the rapid development of network and information technology, people’s access to various kinds of information is not limited to traditional media such as newspapers, television, radio, etc., but more use of online media. The social media operation platform is a computer or mobile client that substantially simplifies and enriches how and what information is accepted by the audience. When large-scale public emergencies occur, an increasing number of people opt to use the media to gather or disseminate various details about the situation. This circumstance can quickly lead to network public opinion, a mix of real and incorrect information, making it difficult to regulate and foresee the event’s development trend. When people pick media intermediary as a route for appeal, they primarily seek to use the media intermediary’s leading role to draw the attention of government departments, so that the public and government departments can have a dialog atmosphere in which to address problems fairly. The public sees the media as a link between the government and the people. As a result, regardless of whether it is traditional official media or online media, event information should be communicated swiftly, truthfully, accurately, and comprehensively following the incident, as the party and the government prevail. The media should be able to accurately predict public opinion, distribute information on schedule, engage with the public, and please the public. The responsibility and obligation of online media, particularly key websites, key forums, and social software, should combat the spread of rumors and pseudo-information and use the characteristics of fast dissemination and authoritativeness of official media to coordinate the release of authority by government departments. The message is to achieve a successful mix of traditional and online media, correctly lead public opinion on the internet, and promote social harmony and stability.

Emotional factors have a direct effect path coefficient of (*b* = 0.27) on behavioral decision-making. Emotional factors can also indirectly influence behavioral decision-making through media intermediary variables. The path coefficient is (*b* = 0.078), and it is positively significant (*P* < 0.001). The total effect of factors on behavioral decision making is (*b* = 0.346). It is easy to see how emotion plays a significant role in developing large-scale public events. Furthermore, emotional factors are the outcome variables of predisposing factors and government credibility. On the other hand, emotional factors are the causal variables in behavioral decision-making. Emotions are a common psychological phenomenon that has a constant impact on people’s lives. Emotions have been shown to have a significant impact on individual decision-making outcomes, decision-making processes, and even decision-making quality, according to studies. Positive (such as optimism emotions) social emotions can be caused by high public sentiment and social development, whereas negative (such as pessimistic) social emotions will endanger social stability if they are not channeled and resolved in a timely manner, especially in specific decision-making situations such as group public emergencies.

### Managerial Implications

Despite internationally known research institutions and governments developing public emergency management, China has promulgated a series of public emergency laws, regulations, and plans. Furthermore, in the face of sudden occurrences of group events and rapid dissemination of information, particularly for destructive events, the public’s negative emotions easily erupt in the group, infect and spread, prompting the public to engage in irrational behavior. On the other hand, they are more difficult to control because of a lack of emergency preparedness and skills for group public emergencies. Emotional factors influence how people behave and make decisions. The two factors are the government’s public reliability factors and the group’s public emergency inducement factors. Endogenous factors of group public emergencies include inducement factors, while exogenous factors include government public reliability. In terms of inducing factors, relevant government departments should rationally examine the occurrence of group public emergencies and recognize that they result from maximizing the interests of various interest groups and the embodiment of a public social fairness imbalance. Relevant government departments are critical for both parties to coordinate and control the situation’s development in terms of government credibility. They are supposed to relieve social tension and antagonism while also acting as a social safety valve. Furthermore, public sentiment does not remain constant throughout the occurrence and evolution of mass emergencies and has different characteristics at different stages.

First and primarily, the causes and details of huge public catastrophes were unclear before their occurrence. The majority of the population is focused on viewing, gathering, and sorting information about the event. As a result, the public mood is usually steady, but this does not rule out the possibility of abnormal emotions, panic, anxiety, or other emotional states in particular individuals or groups. In the final stages of the event, the emotions of these individuals or groups will be crucial. Second, when a large-scale public emergency occurs, the public mood is volatile, and it will spontaneously erupt based on a few fuses. The public’s early comprehension of events and the emotional infection of a few exceptional persons or groups causes the public’s emotions to have the same emotional motive, direction and intensity, making mass public emergencies and irrational collective action more likely. Finally, the core conflicts and contradictions have been resolved appropriately. The public mood has progressively calmed down to achieve a generally stable state in the stage of group public emergencies settling down. It’s worth mentioning that the public mood isn’t completely constant compared to the mood before the election. The public mood is more likely to change at this time. If there are any stimulating events or information, the public mood can easily erupt again, resulting in large public emergencies. The consequences of a reoccurrence of events are more serious than those of the first, and the evolution of events is more difficult to forecast and control. As a result, from the government’s standpoint, and in light of shifting public attitude and the evolving characteristics of large public catastrophes, To interfere in the public’s emotions in three ways: prevention and monitoring, guiding and control throughout the process, and post-stabilization.

To a certain extent, master the decision-making process, power, and critical points of mass public emergencies in order to propose reasonable countermeasures in light of inevitable factors such as inducing factors, government public reliability, and media intermediaries, to prevent and control the occurrence and evolution of mass public emergencies. It is beneficial to investigate the public’s emotional decision-making tendency and ability in response to various public emergencies to improve the public’s emotional decision-making tendency and response to public emergencies; effectively prevent and control the emotional outbreak of group public emergencies.

### Limitations and Future Research Directions

This study examines the primary drivers influencing public behavior decisions in the aftermath of mass occurrences using surveys of a wide population, presents the creation of effective preventative and response tactics and a model of the factors that impact public behavior decision-making. The finding is innovative, however it does have certain flaws that can be addressed and improved in future research. First, this study focuses on the inevitable elements impacting public behavior decision-making following group public emergencies while creating the structural equation model of multi-factor influence on public behavior decision-making under group public emergencies. Factors like social cognition and social background can be integrated into the model in the future for more in-depth investigation. Second, when examining the impact of emotion on public behavior decision-making during group public emergencies, intermediate media elements are considered in this study. Future research can focus on media factors and prominent opinion leaders in group public emergencies, given the media’s increasingly essential guiding role in public opinion. Third, the existing data is exclusively from China, and the majority of the respondents are between the ages of 20 and 40. Comparative investigations will be possible in the future. On the one hand, it is possible to compare whether the data of the young and old in decision-making under group public emergencies will yield different results; on the other hand, data from other countries and regions can be collected for cross-national comparison to further enrich and improve the research conclusions.

## Data Availability Statement

The original contributions presented in the study are included in the article/supplementary material, further inquiries can be directed to the corresponding author/s.

## Author Contributions

NG and RS: conceptualization and formal analysis. CL and NG: methodology. RS: software. NG and CL: validation. NG and RS: investigation. NG: resources. NG: data collection, and writing – original draft preparation. RS: supervision, and writing – review and editing. All authors contributed to the article and approved the submitted version.

## Conflict of Interest

The authors declare that the research was conducted in the absence of any commercial or financial relationships that could be construed as a potential conflict of interest.

## Publisher’s Note

All claims expressed in this article are solely those of the authors and do not necessarily represent those of their affiliated organizations, or those of the publisher, the editors and the reviewers. Any product that may be evaluated in this article, or claim that may be made by its manufacturer, is not guaranteed or endorsed by the publisher.

## Footnotes

1
http://wenjuan.com

## Appendix

**TABLE A1:** Questionnaires items of all study variables.

Item number	Latent variable	Observation variable	Problem description
Q1	Emotional factor	Personal emotion	Company practices have a big impact on my mood
Q2		Group emotion	Company practices have a great impact on the emotions of colleagues
Q3		Key person emotions	The mood of the union chairman or department manager to deal with this matter has a great impact on me.
Q4	Predisposing	Group structure	Colleagues take the lead in taking action, which will make everyone more inclined to participate.
Q5		Group size	The more colleagues you participate, the more likely you are to successfully resolve the issue.
Q6		Group behavior	When a conflict occurs, the behavior of colleagues has a great influence on me.
Q7		Interest expectation	Paying labor and earning wages are very different from what I think.
Q8		Fair	Corporate practices are unfair to me.
Q9	Government credibility	Government credibility	The government management department cannot handle this matter well and can only solve it on its own.
Q10		Legal restraint	It is more difficult to solve similar incidents through legal channels.
Q11	Behavior decision	Altruistic behavior	I will help my colleagues to deal with the problem together until things are resolved.
Q12		Hard struggle	I will defend my rights, even if there is a dangerous conflict.
Q13		Compromise cooperation	I will remain silent and try not to participate.
Q14	Media intermediary	Network new media	If there are new media such as Weibo and WeChat, the matter will be solved as soon as possible.
Q15		Traditional media	If there is an official media such as TV or newspaper, things will be resolved as soon as possible.
